# Effect of Root Coverage on Oral Health Impact Profile (G49): A Pilot Study

**DOI:** 10.1155/2010/252303

**Published:** 2010-06-15

**Authors:** Ulrich Hansmeier, Peter Eickholz

**Affiliations:** ^1^Private Practice, 44287 Dortmund, Germany; ^2^Department of Periodontology, Center for Dental, Oral, and Maxillofacial Medicine, Johann Wolfgang Goethe-University Frankfurt am Main, 60590 Frankfurt am Main, Germany

## Abstract

*Purpose*. The aim of this prospective longitudinal clinical pilot study was the evaluation of the effect on the Oral Health Impact Profile (OHIP) and patient-centered results of the envelope technique for Connective Tissue Graft (CTG). *Methods*. Sixteen patients (11 females) 24 to 71 years of age (42.6 ± 11.1) received CTG that had been harvested from the palate and grafted using the envelope technique. Prior to and 3 months after surgery, all patients were examined clinically, completed the OHIP-G49 questionnaire, and were asked to judge the results of surgery. *Results*. Mean baseline recession depth of 2.5 ± 0.8 mm was reduced by 1.2 ± 0.9 mm (*P* < .001). Root coverage amounted to 48 ± 39%. In 5 of 16 defects complete root coverage was achieved. Pain at the donor site was more pronounced than at recipient site regarding prevalence (8/6; *P* = .007), intensity (2.1 ± 2.3/1.1 ± 1.9 [visual analogue scale]; *P* = .016), and duration (1.4 ± 2.3/0.8 ± 1.4 days; *P* = .042). Baseline OHIP (15.7 ± 12.1) was decreased by 3.6 ± 8.5 three months after surgery (*P* = .139). Thirteen patients (81%) would undergo CTG surgery for similar reasons again. *Conclusions*. Root coverage using CTG according to the envelope technique provided improvement of OHIP as early as 3 months after surgery. Over all, patients were reasonably satisfied with the surgical technique and its results.

## 1. Introduction

Exposed root surfaces in the anterior region may have several consequences: impaired aesthetics, increased risk of root caries, and hypersensitivity. Whereas circularly denuded root surfaces (facial/oral and interproximally recessions) due to periodontitis respond neither completely nor predictively to surgical coverage attempts [1], there are several techniques that are successfully used to treat facial recessions [2]: for example, coronally advanced and lateral positioned flaps, free gingival and connective tissue grafts (CTG), and guided tissue regeneration [3]. CTG according to the envelope technique [4] is an established method with favorable long-term stability [5]. Aesthetics is the main reason given by most patients for root coverage [5]. Although a decent number of clinical studies investigating and comparing the efficacy of different root coverage techniques do exist [3, 6], data on patient-centered outcomes are scarce. However, for a technique aiming at aesthetical improvement patient-centered outcomes, that is, the question whether a patient actually notices aesthetic improvement should be regarded as main outcome variables [5]. Thus, structured reviews addressing root coverage techniques have explicitly stated that clinical trials addressing patient-centered outcomes (aesthetics and postsurgical morbidity) are required [3, 6]. 

The concept of oral health-related quality of life (OHQoL) may be an approach to address patient-centered outcomes. The OHIP (Oral Health Impact Profile) questionnaire is one of several instruments which have been developed to measure OHQoL. It is widely used in clinical research. The 49-item version (OHIP-49) is the most comprehensive questionnaire to assess OHQoL and able to measure patients' problems and symptoms [7]. It is well suited to characterize oral problems and symptoms because the questionnaire items were developed through interviews with patients. As a result, it is a standardized, internationally accepted instrument which was translated into several languages (e.g., German: OHIP-G49). 

Hence, the objectives of this prospective longitudinal clinical pilot study were the evaluation of the effect on the Oral Health Impact Profile (OHIP) [7] and patient-centered results of the envelope technique for CTG.

## 2. Materials and Methods

### 2.1. Patients

All patients, for whom root coverage in Miller class I and II recessions was arranged at the dental office of Dr. Ulrich Hansmeier (UH), were recruited for this study. The following inclusion criteria had to be fulfilled:

- denuded root surface of Miller class I or II,
- age ≥18 years,
- absence of probing pocket depths (PD) ≥ 5 mm at the recession tooth and its adjacent teeth,
- oral hygiene instructions and effective individual oral hygiene (interproximal space plaque index [API] < 35%) prior to surgery [8],
- written informed consent.

Exclusion criteria:

- Miller class III and IV,
- presence of probing pocket depths (PD) ≥ 5 mm at the recession tooth and its adjacent teeth,
- pregnancy,
- hemorrhagic disease, anticoagulative therapy.

The study had been approved by the Institutional Review Board for Human Studies of the Medical Faculty of the Johann Wolfgang Goethe-University Frankfurt am Main (Approval number 320/07).

### 2.2. Clinical Examinations

Immediately prior to surgical therapy the following parameters were assessed at 6 sites per tooth: plaque (present/absent) and probing pocket depths (PD) to the nearest 0.5 mm using a manual periodontal probe (PCPUNC15, HuFriedy, Chicago, USA). At the facial aspect of the test tooth the following parameters were measured to the nearest 0.5 mm using the periodontal probe:

- Recession depth (RD), measured from the gingival margin to the cemento-enamel junction (CEJ).
- Recession width (RW). The periodontal probe was oriented horizontally and located at the most apical convexity of the CEJ. Then, the horizontal distance between the mesial and distal gingival margin was assessed.
- Gingival width (GW), after staining with 3% iodine solution from the gingival margin to the mucogingival border [9].

**Figure 1:**
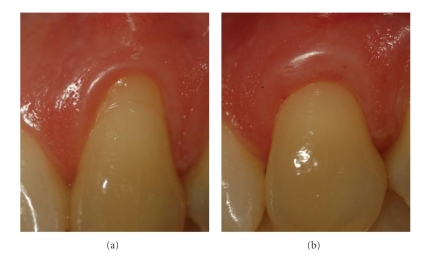
Left maxillary canine (patient #4): (a) before, (b) 3 months after connective tissue graft. The denuded root surface was covered completely. Gingiva is a bit too thick, but the color blends in perfectly.

**Figure 2:**
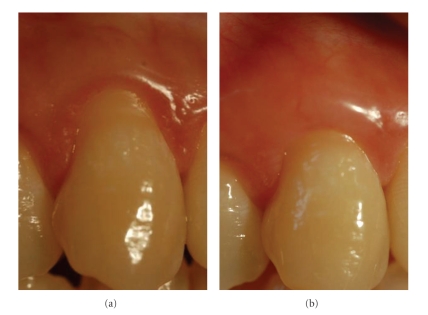
Right maxillary canine (patient #13): (a) before, (b) 3 months after connective tissue graft. The denuded root surface was covered partially.

**Figure 3:**
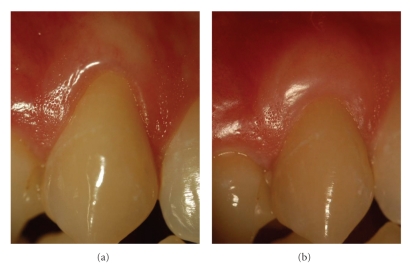
Right maxillary canine (patient #19): (a) before, (b) 3 months after connective tissue graft. The denuded root surface was covered completely. Gingiva is a bit too thick and the color is too light.

Three months after surgical root coverage these measurements were repeated. Root coverage was assessed as reduction of RD. Immediately prior and 3 months after surgery images of the facial aspect of the test teeth were taken (Figures 1–3).

### 2.3. Patient Centered Outcomes

Immediately prior to surgery patients were asked about their personal reason for undergoing surgical root coverage:

- aesthetics,
- hypersensitivity,
- root caries,
- orthodontic therapy.

Immediately prior to and 3 months after surgical therapy patients were asked to complete the OHIP questionnaire (OHIP-G49 clinic version) [7]. 

One week ± 1 day after surgery patients were asked about postsurgical pain.

- Pain intensity after surgery for CTG donor and recipient site were assessed separately using a visual analogue scale (VAS).
- Pain duration (days) after surgery for CTG donor and recipient site separately.
- Number of pain killers taken.

Three months after surgery all patients were questioned as of their opinion and impression of the performed CTG [5]

- Would the patient undergo this kind of surgery again (yes/no).
- By how much has the stated reason of surgery (CTG) improved (school grades: A [very good] to F [insufficient]).
- Satisfaction with the result (grade A [very good] to F [insufficient]).

### 2.4. Periodontal Surgery

The surgical technique that was examined in this study has been described in detail before [4], therefore only a brief description is provided. Local anesthesia was rendered as major palatinal nerve block at the palatal donor region and as infiltrations (Ultracain D-S forte, Sanofi/Aventis, Hoechst, Deutschland) at the buccal aspect of the recession tooth. The denuded root surface was scaled and planed thoroughly until the surface was hard and smooth as probed by an explorer. By removing a tissue collar of 0.5 mm width around the recession the sulcus epithelium was excised. The resulting wound should facilitate vascularisation of the CTG particularly in the gingival margin area. Thereafter a pouch (envelope) was prepared using a no.15c or MB69 blade: Gingiva and mucosa were separated from the periosteum to provide nutrition for the CTG from the underlying periosteum and covering soft tissue. At the palate an incision was made 2 mm paramarginally in the premolar and first molar region. A second parallel incision was made 1 to 2 mm apart from the first, both their mesiodistal length measuring twice the recession width. Both incisions converged into the palatal tissue to meet at the periosteum and, thereby, providing a tissue wedge that was removed from the palate. Thereafter, the epithelium was removed and the CTG was placed within the pouch covering the denuded root surface totally, while being at least 50% submerged within the pouch at the same time. The CTG was fixed with tissue adhesive (Histoacryl, B. Braun Melsungen AG, Melsungen, Germany), and the surgical site was covered by periodontal dressing (Coe-Pak, GC America Inc., Alsip, IL, USA). The harvesting site at the palate was sutured (Permilene 6/0 DSMP13, B. Braun Melsungen AG, Melsungen, Germany). 

Patients were instructed to refrain from mechanical plaque control at the surgical sites for 1 week after surgery. To prevent postsurgical infection, they rinsed with a 0.2% chlorhexidine gluconate solution (Corsodyl, Fink GmbH, Herrenberg, Germany) for 2 minutes, twice daily for this period. Patients were instructed to take pain killers if needed. Ibuprofene was recommended if allergies were excluded. Patients were not provided with prescriptions for pain medications and additional anti-inflammatory drugs were not recommended. Periodontal dressing and sutures were removed 1 week after surgery.

### 2.5. Statistical Analysis

The patient was defined as the statistical unit. Only one recession per patient was included into analysis. If more than 1 recession was treated, the recession with the largest depth was included in all cases. 

The primary end point in this study was chosen to be the change of Oral Health Impact Profile (OHIP-G49) from baseline to 3 months after surgery. Secondary end points were 3 variables used to describe root coverage.

- Absolute reduction of recession height in mm (difference of pre and postsurgical recession height).
- Relative root coverage in % (RD reduction divided by baseline RD multiplied by 100).
- Amount of recession defects showing 100% root coverage postsurgically (number of recession defects with total coverage divided by total number of defects multiplied by 100).

Further secondary end points were 2 variables, used to describe patient-centered outcomes: (i) postsurgical pain, and (ii) patients' judgment of treatment results. 

Prevalence of plaque at facial sites was assessed as number and amount of all sites in per cent. Comparison was made using Wilcoxon sign rank test (two-tailed tests). *P* values were not adjusted for multiple testing. Mean ± standard deviation of baseline and postsurgical PD, recession depth, width, and width of gingiva as well as their difference were calculated and compared using a paired *t*-test with *P* < .05 allowing for statistically significant difference (two-tailed tests). Relative root coverage in % was calculated for each recession defect and mean ± standard deviation for the total sample. Means ± standard deviations were calculated also for patient-centered parameters at baseline, 3 months after surgery and change after 3 months (OHIP-G49) and 1 week after surgery (pain prevalence, intensity [VAS], and duration [days] at donor and recipient site). Comparisons were made using the paired *t*-test (OHIP-G49) and Wilcoxon sign rank test (pain parameters) (two-tailed tests). A number of individuals who judged improvement of the “cause for treatment” and general satisfaction with the treatment result were compared between the group who would and would not undergo surgery again using a *χ* ^2^ test. 

Statistical analysis was performed using a PC program (Systat for Windows version 10, Systat Inc., Evanston, IL, USA).

## 3. Results

### 3.1. Patients

A total of 16 patients (42.6 ± 11.1 years) participated in this study: 5 males with a mean age of 38.0 ± 5.2 years, 11 females with a mean age of 44.7 ± 12.6 years. Surgery took place from February to May 2008. The last re-examination at 3 month was performed in August 2008. The majority of recessions included into the study were Miller class I (14). Two Miller class II lesions were also treated. Most patients aimed for root coverage due to root sensitivity (11) and aesthetics (9). Five patients gave both reasons. Two patients asked for root coverage due to caries, 1 of whom gave root sensitivity as the only reason. Only 1 patient was treated due to occurrence of recession in the course of orthodontic therapy. This patient also complained about hypersensitivity.

### 3.2. Clinical Parameters

At baseline plaque control was highly effective. However, the prevalence of plaque had increased significantly 3 months after surgery. PD was maintained shallow and stable from baseline to 3 months after surgery. Mean presurgical recession depth of 2.5 ± 0.8 mm (1.5–4 mm) was statistically significantly reduced by 1.2 ± 0.9 mm (*P* < .001) to postsurgical 1.3 ± 1.3 mm. Mean presurgical recession width of 4.3 ± 0.9 mm (3.5–7 mm) was statistically significantly reduced by 1.6 ± 1.7 mm (*P* = .003) to postsurgical 2.7 ± 2.1 mm. Mean baseline width of gingiva was 3.3 ± 1.7 mm (0–6 mm) which was statistically significantly increased by 1.5 ± 1.4 mm (*P* < .001) to postsurgical 4.8 ± 1.2 mm. Clinical re-examination revealed a mean root coverage of 48 ± 39%. In 5 of 16 defects (31%) complete root coverage (Figures 1 and 3) had been achieved 3 months after surgery (Table 1).

### 3.3. Patient Centered Outcomes

Eight patients (50%) reported postsurgical pain at the donor site that averaged to an intensity of 2.1 ± 2.3 VAS. Pain at the donor site lasted on average of 1.4 ± 2.3 days. At the recipient site only 6 patients (38%) reported postsurgical pain averaging to an intensity of 1.1 ± 1.9 VAS and duration of 0.8 ± 1.4 days. Pain experience at the donor site was more pronounced than at recipient site regarding all assessed parameters: prevalence (*P* = .007), intensity (*P* = .016), and duration (*P* = .042) (Table 2). At baseline a mean OHIP-G49 of 15.7 ± 12.1 (3–45) was assessed. This mean value was statistically insignificantly (*P* = .139) decreased by 3.6 ± 8.5 (−10–18) 3 months after surgery to 12.1 ± 10.0 (1–35). Only 3 of the total 16 (19%) patients required pain medication after surgery. All of them took just one dose of ibuprofene (200 mg) and reported postsurgical pain either at the donor and the recipient site. The 3 patients taking pain killers reported the highest scores for intensity (donor: 5–7, recipient: 2–6.5) and duration (donor: 3–8 hours, recipient: 2–5 hours) of pain. Thirteen of 16 patients (81%) would undergo CTG surgery for similar reasons again. Within the group, who would not undergo surgery again, the number of individuals who did observe less improvement of the “cause for treatment” (*P* = .139; Table 3) or are generally less satisfied with the treatment result (*P* = .085; Table 4) is larger than that in the group who would undergo surgery again. However, these differences are statistically not significant.

## 4. Discussion

Compared to most studies on root coverage after CTG the reported 1.2 ± 0.9 mm of absolute and 48% of relative coverage are less favorable. Roccuzzo et al. reported between 2.2 and 3.47 mm absolute and between 64.7 and 94.58% relative root coverage after CTG [3]. However, the observation period has to be considered when comparing the results of the present study. The studies referred to by Rocuzzo et al. had to report observation periods of at least 6 months by definition of inclusion criteria. The present study reports results 3 months after surgery, which may be looked upon as too early. Harris had reported a mean creeping attachment of 0.8 mm from 4 weeks after surgery to the final postoperative visit. The mean time between surgery and final postoperative visit was not given. The figures give intervals between 37 and 47 weeks. Creeping attachment occurred in 21 of 22 defects and resulted in complete root coverage in 17 of 22 defects [10]. It may be speculated that root coverage may further increase from 3 to 6 or 12 months after surgery. The short observation period may explain the comparatively small amount of root coverage. However, despite incomplete root coverage 3 months after surgery patient-centered parameters (e.g., OHIP) are already improved. 

More patients reported postsurgical pain at the donor site (50%) than at the recipient site (38%). Further, those, who reported postoperative pain, described it as more intense and longer lasting at the donor (2.1 ± 2.3 VAS; 1.4 ± 2.3 days) than at the recipient site (1.1 ± 1.9 VAS; 0.8 ± 1.4 days). It seems that harvesting of the CTG causes more morbidity than grafting itself. This was reported also in a retrospective analysis, which found none of the 20 patients remembering discomfort after surgery at the CTG recipient site, whereas 4 patients complained about discomfort at the palatal donor site for several days. One patient experienced paraesthesia that lasted for several years [5]. The however rare occasion of palatal sensory dysfunction at the CTG donor site has been described recently [11]. 

The results of this present study did show that root coverage using CTG according to the envelope technique improved the oral health impact profile although the improvement was not statistically significant. To our best knowledge this is the first clinical trial using an OHIP questionnaire to assess patient-centered result of plastic periodontal surgery. These analyses are preliminary due to the small sample and short observation period. However, the study provides data needed to calculate minimal sample sizes for clinical trials to evaluate the differences of oral health impact of different therapies. To detect a mean difference of 3.5 with a test power of 80% and a type 1 error *α* < 0.05 a minimal sample size of 45 sets of defects shall be recruited. In most cases surgery to attain root coverage is performed to improve aesthetics or to reduce sensitivity [5]. Whereas there exists a decent number of clinical studies investigating and comparing the efficacy of different techniques with regard to millimeters or percentages of root coverage [3, 6], data on patient-centered outcomes are scarce. However, for a technique aiming to aesthetical or sensitivity improvement patient-centered outcomes, that is, the question whether a patient actually notices aesthetic or sensitivity improvement, should be regarded as main outcome variables [5]. Thus, future studies should compare root coverage techniques regarding patient-centered outcomes using questionnaires, for example, OHIP. It may be, that patients' satisfaction is achieved by techniques different from those that most favorably improve millimeters and percentages of root coverage. The method of choice to improve aesthetics might not be the method of choice to cover denuded roots completely. 

In the present study for most patients root coverage was performed due to root sensitivity (11) and aesthetics (9). Five patients gave both reasons. In a former questionnaire 16 of 20 patients had asked for root coverage to improve aesthetics, only 4 due to hypersensitivity. In this former trial 19 of 20 patients (95%) would undergo CTG surgery for similar reasons again [5] whereas in this trial only 13 of 16 patients (81%) would undergo surgery again. What may explain this difference in satisfaction after the same type of surgery? Rossberg et al. asked patients retrospectively 6 to 22 years after surgery. The longer the period that has passed after surgery, the higher the likeliness that the patient's memory of discomfort and pain has faded. Three months after surgery as in this analysis, the memory of surgery may still be quite vivid and, thus, more reluctance present to have surgery again. 

Under the limitations of the present study we draw the following conclusions.

- Root coverage using CTG according to the envelope technique provides improvement of OHIP as early as 3 months after surgery.
- Over all, patients were reasonably satisfied with the surgical technique and its results.

## Figures and Tables

**Table 1:** Defect characteristics.

	Baseline	3 Months	Change	*P*
Plaque Index	0.0 ± 0.0	0.3 ± 0.4	0.3 ± 0.4	.046
Probing Pocket Depth/mm	1.4 ± 0.4	1.3 ± 0.4	0.1 ± 0.5	.456
Recession				
Depth/mm	2.5 ± 0.8	1.3 ± 1.3	−1.2 ± 0.9	<.001
Width/mm	4.3 ± 0.9	2.7 ± 2.1	−1.6 ± 1.7	.003
Gingiva/mm	3.3 ± 1.7	4.8 ± 1.2	1.5 ± 1.4	<.001

Relative Root Coverage/%			48 ± 39	

Amount of Complete Root Coverage/n (%)			5 (31)	

**Table 2:** Postsurgical pain.

	Donor site	Recipient site	*P*
Frequency/n (%)	8 (50)	6 (38)	.007
Intensity (VAS)	2.1 ± 2.3	1.1 ± 1.9	.016
Duration/days	1.4 ± 2.3	0.8 ± 1.4	.042

**Table 3:** How much has the reason of surgery (CTG) improved?

School grades	Patient would undergo surgery again	
	No	Yes
A (very good)	0	3
B	2	8
C	0	2
D	2	0
E	0	0
F (insufficient)	1	0

**Table 4:** Satisfaction with treatment result.

School grades	Patient would undergo surgery again	
	No	Yes
A (very good)	0	6
B	2	5
C	0	2
D	1	0
E	0	0
F (insufficient)	0	0
